# Modulation of the input–output function by GABA_A_ receptor-mediated currents in rat oculomotor nucleus motoneurons

**DOI:** 10.1113/jphysiol.2014.276576

**Published:** 2014-10-14

**Authors:** Julio Torres-Torrelo, Blas Torres, Livia Carrascal

**Affiliations:** Department of Physiology, University of SevilleSeville, Spain

## Abstract

The neuronal input–output function depends on recruitment threshold and gain of the firing frequency–current (*f–I*) relationship. These two parameters are positively correlated in ocular motoneurons (MNs) recorded in alert preparation and inhibitory inputs could contribute to this correlation. Phasic inhibition mediated by γ-amino butyric acid (GABA) occurs when a high concentration of GABA at the synaptic cleft activates postsynaptic GABA_A_ receptors, allowing neuronal information transfer. In some neuronal populations, low concentrations of GABA activate non-synaptic GABA_A_ receptors and generate a tonic inhibition, which modulates cell excitability. This study determined how ambient GABA concentrations modulate the input–output relationship of rat oculomotor nucleus MNs. Superfusion of brain slices with GABA (100 μm) produced a GABA_A_ receptor-mediated current that reduced the input resistance, increased the recruitment threshold and shifted the *f–I* relationship rightward without any change in gain. These modifications did not depend on MN size. In absence of exogenous GABA, gabazine (20 μm; antagonist of GABA_A_ receptors) abolished spontaneous inhibitory postsynaptic currents and revealed a tonic current in MNs. Gabazine increased input resistance and decreased recruitment threshold mainly in larger MNs. The *f–I* relationship shifted to the left, without any change in gain. Gabazine effects were chiefly due to MN tonic inhibition because tonic current amplitude was five-fold greater than phasic. This study demonstrates a tonic inhibition in ocular MNs that modulates cell excitability depending on cell size. We suggest that GABA_A_ tonic inhibition acting concurrently with glutamate receptors activation could reproduce the positive covariation between threshold and gain reported in alert preparation.

## Introduction

An individual ocular motoneuron (MN) starts to fire when the position of the eye reaches a certain threshold position in the pulling direction of the muscle that the motoneuron innervates (on-direction). Above this threshold, MN firing rate increases linearly with eye positions in the on-direction (Fuchs & Luschei, 1970). The slope of this relationship (termed *k*) indicates eye position sensitivity. The recruitment threshold and *k* vary across the MN population, and are positively related in alert preparations: MNs with a higher threshold also have greater *k* (Delgado-García *et al*. 1986; Fuchs *et al*. 1988; Davis-López de Carrizosa *et al*. 2011). The origin of the relationship between threshold and *k* is not fully understood, and it was initially proposed that it could depend on MN membrane intrinsic properties and/or on their afferent inputs (Dean, 1997). Because an inverse relationship between recruitment threshold and the slope of the firing frequency–current (*f–I*) relationship has been found in acute *in vivo* recordings and brain slice preparations, intrinsic membrane properties have been ruled out (Grantyn & Grantyn, 1978; Nieto-Gonzalez *et al*. 2007). Neuronal simulation (Dean, 1997; Hazel *et al*. 2002) and ocular MN deafferentation (Pastor & Gonzalez-Forero, 2003) studies support a crucial role of synaptic input in determining the relationship between threshold and gain. The negative covariation between threshold and gain found in oculomotor nucleus MNs disappears with bath application of glutamate, but a positive covariation was not found (Torres-Torrelo *et al*. 2012). Neuronal simulation studies about how a premotor synaptic drive could generate the firing pattern in ocular MNs propose a major role for inhibitory inputs (Hazel *et al*. 2002).

GABA is the major inhibitory neurotransmitter in the mammalian brain. Anatomical studies have demonstrated that the oculomotor nucleus receives a strong GABAergic synaptic input (Soghomonian *et al*. 1989; de la Cruz *et al*. 1992; Spencer *et al*. 1992; Feldblum *et al*. 1993; Spencer & Wang, 1996; Wentzel *et al*. 1996; Horn *et al*. 2003; Che Ngwa *et al*. 2014). Electrical stimulation of the labyrinth evokes ipsilateral inhibitory postsynaptic potentials that are blocked by GABA_A_ receptor antagonists (Ito *et al*. 1970; Highstein, 1973; Precht *et al*. 1973). When GABA acts on GABA_A_ receptors, postsynaptic inhibitory currents are induced that can be classified as phasic or tonic in nature. Phasic inhibitory currents transiently inhibit neurons and occur when a high concentration of GABA in the synaptic cleft binds to and activates low-affinity postsynaptic GABA_A_ receptors (Farrant & Nusser, 2005). This phasic inhibitory action is fundamental for information transfer (Pouille & Scanziani, 2001) and for synchronizing neuronal networks (Sebe *et al*. 2006; Mann & Paulsen, 2007). Tonic inhibitory currents operate on a much slower time scale and take place in a less spatially restricted manner as they are mediated by high-affinity GABA_A_ receptors located in the extra-synaptic membrane. These receptors are sensitive to low GABA concentrations in the extracellular medium, which can originate from diffusion of the neurotransmitter away from the synaptic cleft and by non-synaptic release (Semyanov *et al*. 2004; Farrant & Nusser, 2005; Glykys & Mody, 2007). Tonic activation of GABA_A_ receptors can play a crucial role in regulating neuronal excitability by setting the threshold for action potential generation and shunting excitatory synaptic input (Ruiz *et al*. 2003; Petrini *et al*. 2004; Kulmann *et al*. 2005). Tonic currents are present in brain structures such as the cerebellum (Brickley *et al*. 1996) and hippocampus (Semyanov *et al*. 2003). Despite this knowledge, GABA-mediated tonic inhibition has not been studied in ocular MNs, and data from other motoneuronal pools are scarce (Castro *et al*. 2011; Numata *et al*. 2012; Chesnoy-Marchais, 2013).

The manner in which inhibitory signals affect neuronal computations can be captured by the input–output function, which relates inhibitory input to action potential generation. The input–output function is characterized by two components: the threshold and the gain. Inhibition can change this relationship in two ways: a shift along the input axis could involve dynamic modifications to both the recruitment threshold of each neuron and the recruitment range for a whole population of neurons, whereas a change in slope (gain change) leads to the amplification or scaling down of the sensitivity of the neuron to changes in its inputs (Mitchell & Silver, 2003; Carvalho & Buonomano, 2009). The present work investigates if GABA-mediated currents could contribute to set the recruitment threshold and gain of ocular MNs and if it could act to produce the functional positive relationship between threshold and *k* reported in alert preparations.

## Methods

This study was carried out in strict accordance with the recommendations in the Guide for the Care and Use of Laboratory Animals of the European Community Directive 2003/65 and the Spanish Royal Act 120/2005. The research protocol was approved by the Committee on the Ethics of Animal Experiments of the University of Seville (permit no. 0724-2009). Wistar rats (15–20 days of age) of both sexes were deeply anaesthetized with sodium pentobarbital (50 mg kg^−1^). Brains were quickly removed and placed in a dissection medium containing cold sucrose–artificial cerebrospinal fluid (ACSF). Transverse sections (thickness 300 μm) that included the oculomotor nucleus were cut on a vibratome (Leica 1200S, Wetzlar, Germany), placed in an ACSF-filled chamber for 30 min at ∼37°C, and then stored at ∼21°C in the same solution until use. The composition of the ACSF (in mm) was as follows: 126 NaCl, 2 KCl, 1.25 Na_2_HPO_4_, 26 NaHCO_3_, 10 glucose, 2 MgSO_4_ and 2 CaCl_2_. For the sucrose–ACSF solution, the NaCl was replaced by sucrose (240 mm). Both ACSF and sucrose–ACSF solutions were bubbled with 95% O_2_–5% CO_2_ (pH 7.4).

### Whole cell patch clamp recordings

Slices were superfused with ACSF (33 ± 1°C, 2 ml min^−1^). The oculomotor nucleus was distinguished under the microscope by its colour beneath the periaqueductal grey matter. MNs were patch clamped under visual guidance using a Nikon Eclipse FN1 microscope (Melville, NY, USA) equipped with infrared-differential interference contrast optics, a 40× water immersion objective, and a Hamamatsu C-7500 camera (Hamamatsu UK, Hertfordshire, UK). MNs were functionally identified by their antidromic activation from the root of the third nerve and by the collision test (Carrascal *et al*. 2006). For current clamp recordings, the patch pipettes (3–5 MΩ) contained (in mm): 120 potassium gluconate, 10 KCl, 10 phosphocreatine disodium salt, 2 MgATP, 0.3 NaGTP, 0.1 EGTA, 10 Hepes, adjusted to pH 7.3 with KOH. For voltage clamp recordings, the patch pipettes contained (in mm): 140 CsCl, 2 MgCl_2_, 0.05 EGTA and 10 Hepes, adjusted to pH 7.3 with CsOH. The osmolality of both intracellular solutions was 280–290 mosmol kg^−1^, adjusted with sucrose. Whole cell patch clamp recordings were carried out using a MultiClamp 700B Amplifier (Molecular Devices, Sunnyvale, CA, USA). Giga seals (>1 GΩ) were always obtained before rupture of the patch. Only recordings with an access resistance between 5 and 20 MΩ were accepted for analysis. Series resistance was routinely compensated by 70%. Current and voltage clamp recordings were low-pass Bessel-filtered at 10 kHz; data were digitized at 20 kHz with a Digidata 1440A analog-to-digital converter and acquired using pCLAMP 10 software (Molecular Devices). Data were stored on a computer disk and analysed offline by using Clampfit 10.2 (Molecular Devices) software.

All drugs were prepared just before experiments from stock solutions stored at −20°C. In general, the protocol used for studying the effects of drugs was as follows: each MN was initially superfused with normal ACSF to obtain baseline voltage clamp control data or to study electrophysiological parameters in current clamp mode under control conditions. After this, to determine the effect of high GABA concentrations, the slice was superfused for 3 min with ACSF containing 100 μm GABA (Sigma, St. Louis, MO, USA) and current or voltage responses were again acquired (GABA conditions). On the other hand, tonic GABA_A_ receptor-mediated currents were revealed by blocking the GABA_A_ receptor with the antagonist drug 2-(3-carboxypropyl)-3-amino-6-methoxyphenyl-pyridazinium bromide [SR95531 (gabazine), 20 μm; Tocris (Bristol, UK)] in a solution containing a low extracellular GABA concentration (i.e. lacking added exogenous GABA). Gabazine was injected directly into the slice chamber and the current and voltage responses recorded. Only one MN per slice was analysed to avoid concerns associated with incomplete reagent washout or incomplete recovery to baseline conditions.

#### Voltage clamp recordings and analysis

Membrane current was recorded from MNs held at −60 mV, and a CsCl-based internal pipette solution was used to obtain an *E*_Cl_ ∼0 mV. The current amplitude evoked by the application of 100 μm GABA was calculated as the difference compared with the baseline level. The amplitude of the GABA_A_ receptor-mediated tonic current was calculated as the difference between the membrane current before and following gabazine injection. The phasic current was calculated by multiplying the charge transfer (measured as area under the averaged spontaneous inhibitory postsynaptic currents) with the synaptic frequency (Nusser & Mody, 2002; Gao & Smith, 2010).

#### Current clamp recordings and analysis

Current clamp experiments were conducted to determine whether the inhibitory current elicited by 100 μm GABA, or block of the GABA_A_ receptor-mediated tonic current, modulates the intrinsic membrane properties of oculomotor nucleus MNs. The following parameters were quantified: resting membrane potentials were measured as the difference between the intracellular and extracellular potentials after withdrawing the recording electrode from the cell. The input resistance was determined by passing negative current steps (500 ms, 1 Hz; with 10 pA increments), and calculated as the slope of the current-voltage plot. The recruitment threshold was measured from rheobase. The rheobase was the minimum current injected (100 ms, 1 Hz; with 10 pA increments) that generated an action potential in 50% of cases. The depolarization voltage was the increase in membrane potential required for the cell to reach the spike threshold. To determine the spike threshold, the action potential recording was differentiated, with the spike onset taken as the value of the membrane potential at which the first derivative exceeded 10 V s^−1^. The tonic component of firing was measured from the repetitive discharge evoked by depolarizing current steps (1 s, 0.5 Hz) with 10–50 pA increments. The steady-state firing frequency was taken as the average number of spikes during the last 500 ms of the repetitive discharge. The relationship between the steady-state firing frequency and injected current was plotted (*f–I* plot) and the slope (gain) calculated.

### Intracellular labelling and reconstruction

To determine if our sample of MNs followed the size principle (Henneman *et al*. 1965; Mendell, 2005), experiments were carried out on 300 μm thick brain slices as follows. The input resistance was recorded from MNs (*n* = 33), which were then intracellularly labelled with neurobiotin (Neurobiotin tracer; Vector Laboratories, Burlingame, CA, USA). This was done by the iontophoretic injection of 1% neurobiotin contained in the internal pipette solution by applying current steps (400–500 pA) of 500 ms at 0.5 Hz for 10–20 min. Slices were then transferred to a solution of 4% paraformaldehyde at 4°C overnight and then to 30% sucrose in phosphate buffer at 4°C overnight. Thereafter, neurobiotin labelling was revealed with monoclonal antibiotin fluorescein isothiocyanate-conjugated antibody (dilution 1:120; Sigma) and visualized with a confocal microscope (Leica TCS SP2, Wetzlar, Germany). Photomicrographs of the MN cell bodies were taken at different depths, 1 μm apart, and then used to reconstruct the cell somata in 3D. Reconstructions and somatic membrane surface measurements were carried out with Imaris software (version 7.3, Bitplane Inc., St. Paul, MN, USA).

### Vesicular GABA transporter immunohistochemistry

Rats (*n* = 3) were perfused with phosphate-buffered saline (PBS) followed by 4% paraformaldehyde in PBS and post-fixed in the same solution for 2–3 h. After overnight incubation in 30% sucrose in PBS, 40 μm thick sections were cut on a cryostat (Leica CM1850). To determine if the oculomotor nucleus received GABAergic inputs, double immunohistochemistry was carried out, with anticholine acetyltransferase (ChAT) antibody used to verify that cells were MNs, and antivesicular GABA transporter (VGAT) used to visualize GABAergic terminals. Sections processed for immunohistochemistry were preincubated free-floating in blocking solution (3% bovine serum albumin) at room temperature for 1 h and then incubated with both primary antibodies – anti-ChAT (goat anti-ChAT affinity purified polyclonal antibody, 1:100; Millipore, Darmstadt, Germany) and anti-VGAT (rabbit polyclonal anti-VGAT, 1:500; Millipore) – in blocking solution at 4°C overnight. Sections were incubated for 2 h with both secondary antibodies (Cy3-conjugated affiniPure donkey antigoat for anti-ChAT, diluted 1:200, and fluorescein isothiocyanate-AffiniPure Donkey Anti-Rabbit IgG for VGAT; Jackson ImmunoResearch, Philadelphia, PA, USA), mounted with fluorescence mounting medium (Dako, Glostrup, Denmark), and visualized on a confocal microscope (Leica TCS SP2).

### Statistical analysis

Significant differences between control and test (in presence of drugs) conditions were determined by using the Student's *t* test for paired samples. The correlation between variables was measured by Pearson's correlation coefficient (*r*). The significance level was established at *P* < 0.05. All data are reported as means ± s.e.m.

## Results

**Figure 1:**
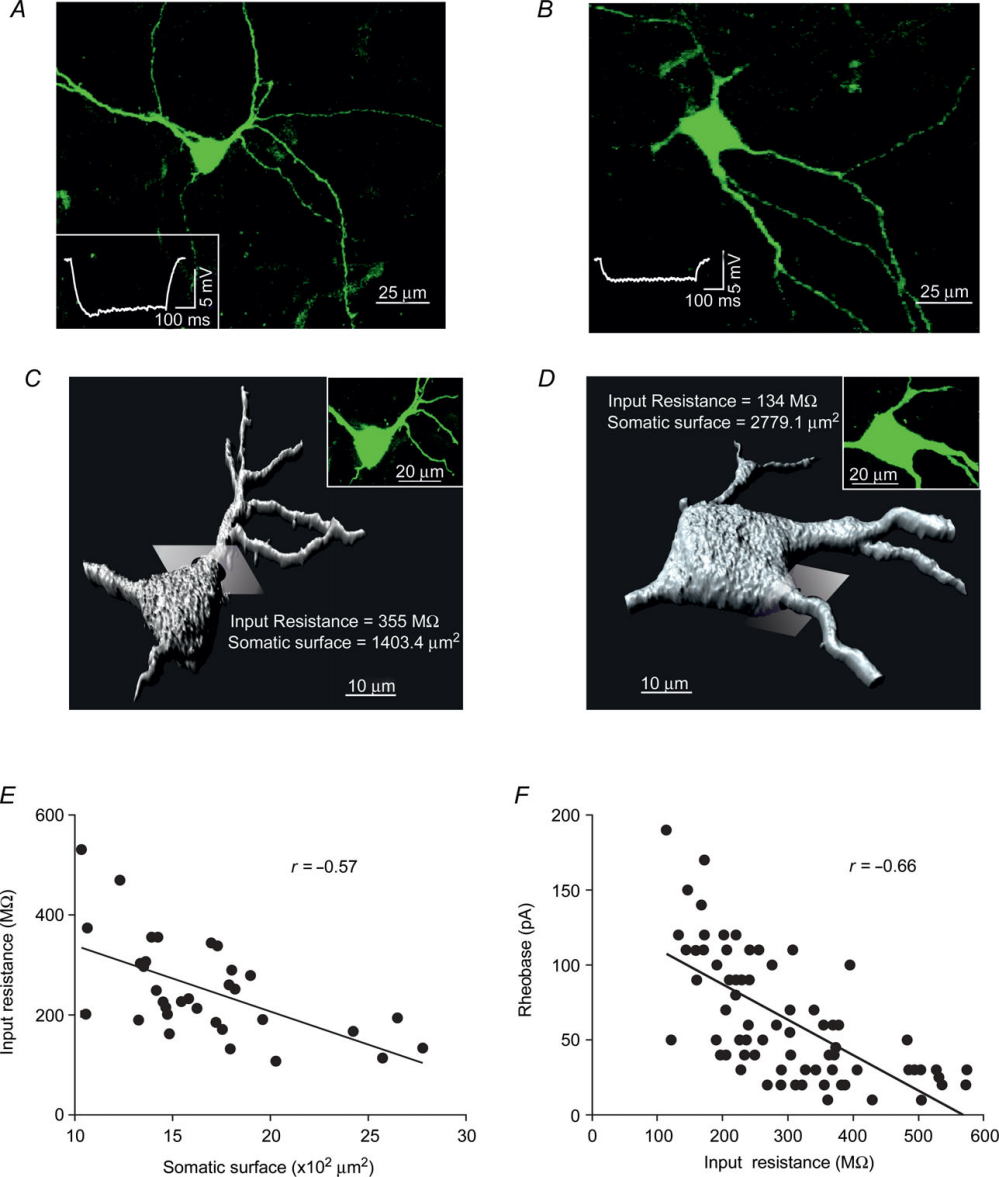
Relationship between cell size, input resistance and rheobase in MNs of the rat oculomotor nucleus *A* and *B*, cell bodies and main dendritic trees of two MNs with input resistances of 355 MΩ (*A*) and 134 MΩ (*B*). Insets in (*A*) and (*B*) show the membrane potential responses to negative current steps (20 pA) of labelled MNs. *C* and *D*, three-dimensional reconstruction of the MNs shown in (*A*) and (*B*), respectively. To obtain the somatic surface values the Imaris software established a separation by a plane between the cell body and main dendrites (one of these planes is shown for each MN). *E*, relationship between somatic membrane surface area and input resistance (*n* = 33). *F*, relationship between input resistance and rheobase (*n* = 73). Linear correlations are illustrated. MNs, motoneurons.

According to the size principle, the order of recruitment of MNs is considered to depend on their input resistance, such that smaller MNs with less surface area would have higher input resistance and would produce a larger voltage drop for a given synaptic input than larger MNs (Henneman *et al*. 1965; Mendell, 2005). To determine if the effects on intrinsic membrane properties of high concentrations of GABA and/or the blocking of GABA_A_ receptors by gabazine depended on cell size, we studied the relationship between somatic surface area and input resistance, and between input resistance and rheobase. Figure1*A* and *B* provides two representative examples of labelled MNs with input resistances of 355 MΩ and 134 MΩ, respectively. The somatic surface area of these MNs was reconstructed and measured (Fig.1*C* and *D*) to be 1403.4 μm^2^ and 2779.1 μm^2^, respectively. An inverse correlation showing a significant linear relationship between somata surface area and input resistance was found (*n* = 33; Fig.1*E*). Furthermore, as the input resistance of MNs increases, lower currents are required for these MNs to be recruited (rheobase), with a significant linear relationship found between these two parameters (*n* = 73; Fig.1*F*).

**Figure 2:**
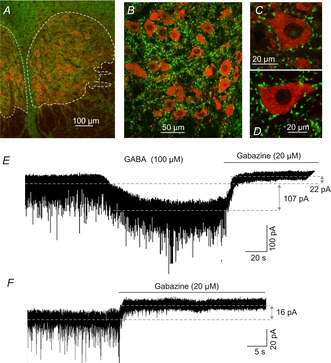
GABA inputs and currents in MNs of the rat oculomotor nucleus *A–D*, vesicular transporter of GABA (VGAT, green) within the boundaries of the rat oculomotor nucleus (delineated by dashed lines, *A*), in the neuropil (*B*), and in close association with MN cell bodies (*C*) and (*D*). MNs were identified by their positive immunoreactivity against choline acetyltransferase (red). *E*, inward current evoked by application of 100 μm GABA. This current (107 pA) was mediated by GABA_A_ receptors as it was blocked by 20 μm gabazine. *F*, gabazine injection (20 μm) blocked the spontaneous inhibitory postsynaptic currents and revealed a tonic current of 16 pA in the illustrated MN. This current can also be seen in (*E*) after gabazine application (22 pA). MNs, motoneurons.

Because GABAergic inputs to the rat oculomotor nucleus are yet to be demonstrated physiologically, we studied the localization of the vesicular GABA transporter, VGAT, in three animals (15, 17 and 20 days postnatal). Inspection of transverse sections of brains in these three cases revealed abundant synaptic terminals in the neuropil (Fig.2*A* and *B*). In addition, we observed a large number of stained terminals in close association with MN cell bodies, which were identified by immunohistochemistry against ChAT (Fig.2*C* and *D*). Consistent with these anatomical observations, GABA (100 μm) application to the bath produced a shift in the inward current carried by Cl^−^ (*E*_CL_ = 0 mV) in all MNs studied (*n* = 8), with the current reaching a plateau level at 129 ± 23 pA (Fig.2*E*). This current was mediated by GABA_A_ receptors as it was blocked by gabazine. To examine if these MNs exhibit a tonic GABA_A_ receptor-mediated current in a low GABA concentration (i.e. lacking exogenous GABA), gabazine injections (20 μm) were delivered into the slice chamber in the presence of kynurenic acid (2 mm) to block glutamatergic currents (*n* = 8). Under these conditions, spontaneous inhibitory postsynaptic currents were blocked, and an outward shift in the holding current was recorded when the holding potential was held at −60 mV (Fig.2*F*). To assess the contribution of phasic and tonic currents on gabazine effects in the MN input–output function, we compared the amplitude of these currents. The frequency of spontaneous inhibitory postsynaptic currents was 2.7 ± 0.61 Hz and the charge transfer for averaged spontaneous inhibitory postsynaptic currents was 0.6 ± 0.09 pC. The mean phasic current amplitude was 1.7 ± 0.5 pA and the mean tonic current amplitude was 9.2 ± 1.5 pA. Therefore, in low GABA concentrations the tonic current was five-fold larger than the phasic current (i.e. 85% of the total GABA_A_ receptor-mediated currents). The effects of GABA and gabazine could be reversed by washing slices with ACSF for 20 min.

**Figure 3:**
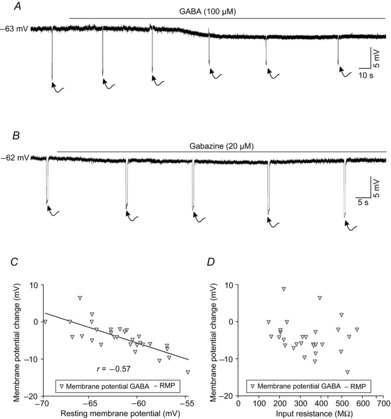
Effects of GABA (100 μm) or gabazine (20 μm) on the membrane potential and input resistance of MNs from the rat oculomotor nucleus *A*, bath application of 100 μm GABA yielded a membrane potential hyperpolarization and a diminution in the membrane response (see arrows) to negative current steps of 50 pA. *B*, gabazine produced an increase in the membrane response (see arrows) to negative current steps of 50 pA, while the RMP remained largely unaltered. *C*, relationship between RMP and membrane potential change evoked by 100 μm GABA. Note that the amplitude of membrane potential change diminished when rest values were close to −67 mV. *D*, relationship between input resistance and membrane potential change. MNs, motoneurons; RMP, resting membrane potential.

As stated in the introduction, membrane hyperpolarization and shunting are the two main mechanisms mediating neuronal inhibition. Figure3*A* and *B* illustrates the effects of GABA and gabazine application on membrane potential and input resistance in two representative MNs. GABA-induced membrane potential hyperpolarization and a decrease in the voltage response to current steps of −50 pA (i.e. a decrease in the input resistance, see arrows in Fig.3*A*). A comparison of the membrane potential values showed a significantly different outcome between control and GABA conditions (Table1). The membrane potential of MNs exposed to GABA drifted to values close to −67 mV. Indeed, an inverse significant relation was found between resting membrane potential and membrane potential change (Fig.3*C*). The farther the resting membrane potential was from −67 mV, the larger of the magnitude of drift was. The effect of GABA on the membrane potential was not significantly related to the input resistance (Fig.3*D*). In contrast to GABA-induced effects, gabazine did not modify the membrane potential, but did increase input resistance (Fig.3*B*).

**Table 1:** Effect of GABA (100 μm) or gabazine (20 μm) on intrinsic membrane properties of the oculomotor nucleus motoneurons

	Experimental group 1 (*n* = 23)		Experimental group 2 (*n* = 28)	
	Control	GABA	Control	Gabazine
Membrane potential (mV)	−63.2 ± 0.7	−66.1 ± 0.6^*^	−61.5 ± 0.9	−60.9 ± 0.9
Input resistance (MΩ)	320.6 ± 27.6	209.9 ± 20.4^*^	284.5 ± 21.0	375.4 ± 21.1^*^
Time constant (ms)	24.2 ± 1.6	16.3 ± 1.4^*^	20.1 ± 0.9	26.5 ± 0.8^*^
Rheobase (pA)	59.3 ± 7.9	129.8 ± 12.4^*^	67.8 ± 8.9	52.1 ± 6.3^*^
Voltage threshold (mV)	−39.1 ± 1.2	−38.5 ± 1.7	−37.7 ± 1.08	−36.4 ± 1.3
Tonic frequency gain (sp nA s^−1^)	187.5 ± 25.9	161.8 ± 18.2	197.4 ± 15.0	175.3 ± 18.3

Statistical differences (*P* < 0.05) are indicated by an asterisk. sp, spikes.

**Figure 4:**
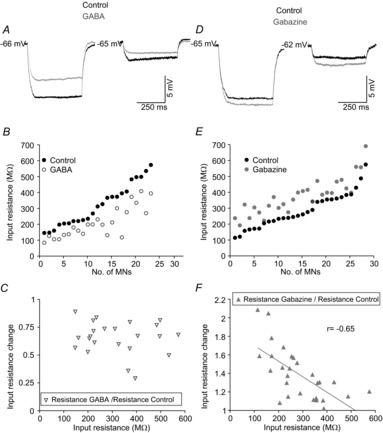
Effects of GABA (100 μm) or gabazine (20 μm) on input resistance of MNs from the rat oculomotor nucleus *A* and *D*, membrane potential responses to current steps of 20 pA in high- and low-input resistance MNs under control conditions and during exposition to GABA (*A*) or gabazine (*D*). *B* and *E*, plots illustrating the input resistance value of each MN under control conditions and during exposure to GABA (*B*) or gabazine (*E*). *C* and *F*, relationship between input resistance under control condition and input resistance change in response to exposure to GABA (*C*) or gabazine (*F*). Note that the input resistance changed linearly with input resistance in response to gabazine exposure, but not with GABA application. MNs, motoneurons.

As expected, a high GABA concentration yielded a significant decrease in MN input resistances (*n* = 23; Table1). Figure4*A* illustrates the effect of GABA (100 μm) on two representative MNs with high- and low-input resistances, respectively, under control conditions. The decrease in input resistance was more pronounced in the high-input resistance MN (from 496.5 to 335.4 MΩ) than in the low-input resistance MN (from 160.2 to 105.8 MΩ) for an equivalent hyperpolarizing current step amplitude (20 pA). However, the relative change (input resistance in presence of GABA/input resistance under control conditions) was similar in both MNs (∼35%). These results are further documented in Fig.4*B* for the whole population of MNs tested. Input resistances ranged from 146 to 573.5 MΩ under control conditions, and from 84 and 408.4 MΩ in the presence of GABA. These results show that, in MNs with high-input resistance (i.e. small MNs), GABA produced a more pronounced decrease in absolute values. However, this size-dependent effect disappeared when data were normalized (input resistance GABA/input resistance control) and plotted as a function of the input resistance under control conditions (Fig.4*C*).

Blocking GABA_A_ receptors with gabazine produced a significant increase in MN input resistance (*n* = 28; Table1). Figure4*D* shows voltage changes in response to hyperpolarizing current steps of 20 pA in a high- and a low-input resistance MN, under control conditions and in the presence of gabazine. The increase in input resistance was similar in the high- (from 487.3 to 557.8 MΩ) and in the low-input resistance MN (from 121.1 to 192.1 MΩ). However, the relative change (input resistance in presence of gabazine/input resistance under control conditions) was higher in the MN with lower input resistance. Consistent with these findings, the raw data illustrated in Fig.4*E* reveals that gabazine produced changes in input resistance whose absolute values did not vary significantly among MNs. In this way, input resistances ranged between 113.9 and 574.9 MΩ for control conditions, and between 192.1 and 690.4 MΩ in the presence of gabazine. The association between input resistance under control conditions (i.e. somata size) and the normalized input resistance change was significantly fitted by an inverse linear relation (Fig.4*F*). Thus, the input resistance of the MNs recruited first (i.e. those with high-input resistance) were almost unaffected by gabazine, whereas input resistance values were doubled in MNs recruited last. This finding supports the hypothesis that extra-synaptic high-affinity GABA_A_ receptors mediate a tonic conductance that depends on cell size (see Discussion).

**Figure 5:**
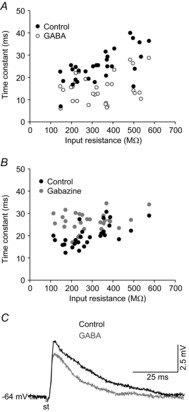
Effects of GABA (100 μm) or gabazine (20 μm) on the membrane time constant in motoneurons from the rat oculomotor nucleus *A* and *B*, time constant value for each motoneuron under control condition and following exposure to GABA (*A*) or gabazine (*B*), with results expressed as a function of the input resistance under control conditions. *C*, representative recordings of postsynaptic excitatory potentials evoked by the stimulation of the medial longitudinal fasciculus under control conditions and during GABA application. st, stimulation.

A high ambient concentration of GABA and the blocking of GABA_A_ receptors by gabazine also evoked changes in the membrane time constant. Modification of this parameter could alter the time window over which synaptic integration occurs (Semyanov *et al*. 2004). Superfusion of brain slices with 100 μm GABA significantly decreased the membrane time constant (Table1), the magnitude of which, as illustrated in Fig.5*A*, was more pronounced in MNs with higher input resistances. In contrast, gabazine significantly increased the membrane time constant in a manner that was more evident in MNs with low-input resistance (Fig.5*B*; Table1). To determine the effect of GABA (*n* = 3) and gabazine (*n* = 3) on postsynaptic potentials, the medial longitudinal fasciculus was stimulated and the response recorded. Figure5*C* shows that the amplitude and time constant of the excitatory postsynaptic potentials decreased during GABA application, whereas they increased in the presence of gabazine (not shown).

**Figure 6:**
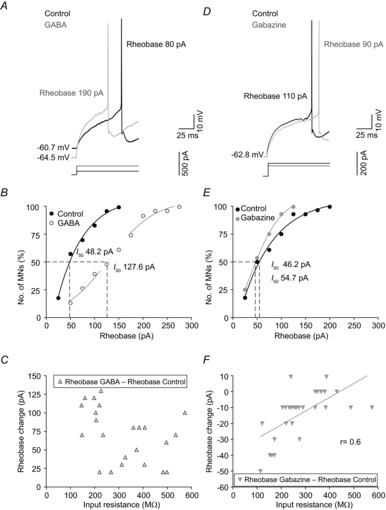
Effects of GABA (100 μm) or gabazine (20 μm) on the rheobase of MNs from the rat oculomotor nucleus *A* and *D*, minimum current (rheobase) required to evoke an action potential under control conditions and in response to GABA (*A*) or gabazine (*D*) in two different MNs. *B* and *E*, cumulative normalized plots of rheobase under control conditions and during exposure to GABA (*B*) or gabazine (*E*). The dashed lines show the rheobase (*I*_50_) values at which 50% of MNs fired off an action potential*. C* and *F*, relationship between input resistance under control conditions and rheobase change in response to exposure to GABA (*C*) or gabazine (*F*). MNs, motoneurons.

To determine if GABA or gabazine-evoked changes in passive membrane properties were sufficient to modify the action potential threshold, we first studied the effects of these compounds on the minimum injected current and voltage needed to generate an action potential (rheobase and voltage threshold, respectively). GABA produced a significant increase in rheobase without causing any change in the voltage threshold (Fig.6*A*; Table1), and a rightward shift in the rheobase was seen when data were plotted in a cumulative normalized diagram (Fig.6*B*). According to this plot, under control conditions 50% of MNs fired for rheobase values ≤48.2 pA, while in the presence of GABA, 50% of MNs fired for rheobase ≤127.6 pA. Moreover, it can be seen from the plot that, under control conditions, 100% of MNs were recruited at a rheobase ≤150 pA, while in the presence of GABA almost 50% of MNs were silent at this current amplitude. We did not find any significant correlation between the magnitude of change in rheobase (rheobase GABA − rheobase control) and input resistance, which implies that the former does not depend on cell size (Fig.6*C*). Regarding the effects of gabazine on the threshold voltage required for an action potential to be elicited, a block of the tonic GABA-mediated current did not produce any significant changes in voltage threshold, whereas a significant increase was measured for the rheobase (Fig.6*D*; Table1). This decrease in rheobase was different among MNs (Fig.6*E* and *F*). As shown in Fig.6*E*, all MNs were recruited at a rheobase value ≤125 pA in the presence of gabazine, while ∼15% of MNs were not recruited at this current under control conditions. However, 50% of MNs were recruited at a rheobase ≤50 pA under both control and gabazine conditions, with no significant shift observed from inspection of the plot (Fig.6*E*). Further to this, the change in rheobase and input resistance under control conditions showed a significant linear relationship (Fig.6*F*). Taken together, these results suggest that the inhibition of GABA_A_ receptors mediating the tonic current produced a more powerful effect upon the rheobase in low- compared with high-input resistance MNs.

**Figure 7:**
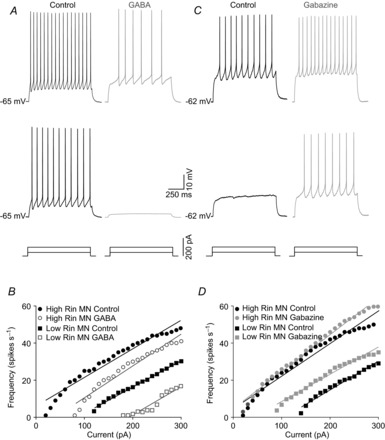
Effects of GABA (100 μm) or gabazine (20 μm) on *f–I* relationships in MNs from the rat oculomotor nucleus *A* and *C*, firing of the same MN in response to two current steps of different intensities under control conditions and in response to GABA (*A*) or gabazine (C). *B* and *D*, *f–I* plots for two representative MN with low- and high-input resistance under control conditions and during exposure to GABA (*B*) or gabazine (*D*). MNs, motoneurons.

Finally, the effects of 100 μm GABA (*n* = 35) or gabazine (*n* = 28) upon the *f–I* relationship was studied. Some MNs (12 of 35) fired only a few initial spikes in response to high intensity current steps (>200 pA) when GABA was present in the bath. For the purpose of this study, MNs that could not sustain a steady-state level of spike firing in response to the current protocol used in the presence of GABA were not included in the analysis. Figure7*A* and *B* illustrates the response of a representative MN to supra-threshold depolarizing current steps under control conditions and in the presence of GABA. In response to current steps of 50 and 100 pA, the MN fired off 11 and 17 spikes s^−1^ under control conditions, while in the presence of GABA the MN did not discharge at the 50 pA current step and only fired at 6 spikes s^−1^ in response to the 100 pA step (Fig.7*A*). Figure7*B* shows the *f–I* relationships for two MNs with different input resistances. GABA application yielded a rightward shift in the *f–I* relationship that was similar for both MNs. Hence, GABA produced an increase in recruitment threshold but no major difference was found in gain. This finding was consistent for the whole population of MNs tested (*n* = 23; Table1).

Figure7*C* illustrates the response of a low-input resistance MN to depolarizing current steps. This MN did not discharge in response to a 50 pA stimulus, and produced 10 spikes s^−1^ in response to a 100 pA current stimulus under control conditions. In the presence of gabazine, the MN discharged at a firing rate of 8 and 15 spikes s^−1^, respectively, in response to the same stimuli intensities. Figure7*D* shows the *f–I* relationships for two MNs with different input resistances. The high-input resistance MNs exhibited similar recruitment thresholds and gains in the *f–I* relationships for control and gabazine conditions. In contrast, low-input resistance MNs showed a leftward shift in the *f–I* relationship, with a modest decrease in gain for the gabazine condition compared with control. For the whole population of MNs (*n* = 28), we found a significant decrease in recruitment threshold with no significant modification in gain (Table1). This finding suggests that gabazine reduces the recruitment threshold, but did not modify gain, as a function of MN size.

## Discussion

This study demonstrates that oculomotor nucleus MNs exhibit a tonic inhibitory current in low ambient GABA concentrations (i.e. where exogenous GABA is lacking). In this ambient GABA, the blockage of GABA_A_ receptors by gabazine caused an increase in input resistance and a decrease in the threshold for spike recruitment as a function of cell size. Brain slice superfusion with 100 μm GABA yielded an inward current that was blocked by gabazine, and shifted the membrane potential to about −67 mV (near the reversal potential for Cl^−^). These two results suggest that the effects of a high GABA concentration are mediated by activation of the GABA_A_ receptors. This suggestion is also supported by the low density of GABA_B_ receptors found in ocular motor nuclei (Margeta-Mitrovic *et al*. 1999) and by the lack of these receptors in the membrane of ocular MNs (Russier *et al*. 2002). However, the contribution of the GABA_B_ receptors and/or the less common GABA_C_ receptors (Chesnoy-Marchais, 2013) cannot be completely ruled out.

Independent of cell size, high ambient GABA concentration reduced input resistance, which caused shorter time constants, and increased MN recruitment threshold without a change in gain. Because low, but not high, concentrations of GABA modulate the input resistance and recruitment threshold as a function of MN size, they must recruit different GABA_A_ receptor pools. We propose that the effects of 100 μm GABA are mainly due to the activation of low-affinity GABA_A_ receptors (i.e. those involved in phasic inhibitory current), while gabazine effects are mainly due to the blockage of high-affinity GABA_A_ receptors (i.e. those involved in tonic inhibitory currents). In agreement with other studies (Semyanov *et al*. 2003; Scimemi *et al*. 2005; Park *et al*. 2006; Gao & Smith, 2010), gabazine effects in oculomotor nucleus MNs were mainly due to tonic inhibition because tonic current accounted for 85% of the total GABA_A_ receptor-mediated current, and tonic inhibition results from the persistent activation of a heterogeneous population of high-affinity GABA_A_ receptors located in the non-synaptic space (Semyanov *et al*. 2004; Farrant & Nusser, 2005; Glykys & Mody, 2007). One hundred micromol per litre of GABA activates both low- and high-affinity GABA_A_ receptors (Mortensen *et al*. 2004; Stórustovu & Ebert, 2006), but the latter are less abundant than their low-affinity counterparts are (Nusser *et al*. 1995). Therefore, it could be suggested that the weight of tonic but not phasic inhibition modulates input–output function depending on cell size in oculomotor nucleus MNs.

### Effects of GABA_A_ receptor-mediated inhibition of gain in the frequency–current relationship

It is widely accepted that ambient GABA concentrations play an important role in cell excitability. *In vivo* studies have shown that iontophoretic injections of GABA or gabazine lead to a decrease or increase, respectively, in the firing rate of neurons in response to sensory stimuli (Ingham & McAlpine, 2005; Duguid *et al*. 2012; Duque *et al*. 2014). As stated in the introduction, gain is one of the two components that characterize the input–output relationship of a system. At the single neuron level, gain is defined as the slope of the *f–I* relationship. In the present study, gain was determined by applying constant current steps of different intensities and measuring the firing frequency in MNs exposed to GABA or gabazine. A rightward or leftward shift in the *f–I* relationship was found during GABA or gabazine treatments, respectively, with a non-significant change observed with respect to gain. These results are in agreement with other studies using a similar protocol. The blocking of tonic inhibition in cerebellar granule cells decreases the current required to achieve a given firing rate because the input–output relationship is shifted to the left (Brickley *et al*. 1996; Hamann *et al*. 2002; Chadderton *et al*. 2004); in spinal MNs, the shift in the *f–I* relationship was proportional to the conductance increase, without changing gain (Brizzi *et al*. 2004). Alternatively, the gain of the input–output relationship has been studied by eliciting synaptically driven membrane potential fluctuations (also called synaptic noise) instead of constant current depolarizing steps. With this procedure it was suggested that the overall level of synaptic input to a neuron acts as a gain control signal (Chance *et al*. 2002). Tonic inhibition coupled with excitatory drive produces a modest diminution of gain (Mitchell & Silver, 2003; Rothman *et al*. 2009). Indeed, tonic GABA_A_ receptor-mediated currents have a minimal effect upon subthreshold membrane potential variations due to synaptic noise, and so exclusively affect neuronal recruitment threshold and not gain (Pavlov *et al*. 2009). Taking these data together, it would seem that ambient GABA concentrations do not modify the input–output gain, or only contribute in a subtle manner to the setting of gain.

### Modulation of motoneuron recruitment threshold by GABA_A_ receptor-mediated currents

Skeletal MNs are activated in an orderly sequence to govern muscle contraction. According to the size principle, the order of recruitment of MNs is considered to depend on the input resistance–cell size (Henneman *et al*. 1965, Mendell, 2005). However, recruitment order also varies with the activity patterns of afferent neurons and with synaptic input organization (Heckman & Binder, 1993; Cope & Sokoloff, 1999). The amplitudes of synaptic inputs from different systems have been shown to vary systematically in low- *versus* high-threshold MNs (Heckman & Binder, 1993). For example, Ia excitatory afferent inputs from homonymous muscle spindles generate a more effective synaptic current in low- compared with high-threshold MNs (Heckman & Binder, 1988), whereas rubro-, vestibulo- or corticospinal inputs to MNs have the opposite organization (Powers *et al*. 1993; Westcott *et al*. 1995; Binder *et al*. 1998). Furthermore, excitatory inputs induce greater responses in higher threshold ocular MNs (Broussard *et al*. 1995; Torres-Torrelo *et al*. 2012). The present data show that, under control *in vitro* conditions, the order of recruitment of oculomotor nucleus MNs from juvenile rats is determined by input resistance–cell size and that a high ambient GABA concentration decreases MN excitability irrespective of cell size. These data are consistent with those obtained in spinal MNs following electrical stimulation of Ia-inhibitory synaptic inputs (Heckman & Binder, 1991) or with those obtained in abducens nucleus MNs following inhibitory deafferentation (Pastor & Gonzalez-Forero, 2003). In conclusion, the GABA_A_ receptor-mediated currents, which were activated by a high concentration of GABA, do not disrupt the orderly recruitment of oculomotor nucleus MNs.

The blocking of tonic current by gabazine produced size-dependent effects upon the recruitment threshold of MNs. Low-threshold MNs were relatively unaffected by gabazine, while high-threshold MNs showed a decreased current threshold (by up to ∼25%) necessary to induce firing. Tonic current amplitude is modulated by the level of expression of high-affinity GABA_A_ receptors, whose kinetics are fine-tuned by modulators (Ortinski *et al*. 2006; Walker & Semyanov, 2008); the crucial factor that regulates tonic current amplitude, however, is the extracellular GABA concentration, which in turn is under the control of GABA transporters that take up the neurotransmitter into neurons and glia, thereby dynamically modulating brain excitability (Richerson & Wu, 2003; Kersanté *et al*. 2013). Indeed, *in vivo* microdialysis studies have demonstrated that extracellular GABA can vary three-fold with exposure to new environments (Bianchi *et al*. 2003). In short, the modulation of tonic current by extracellular GABA levels together with the decrease in recruitment threshold as MN size increases could contribute to expand or reduce the range of recruitment and to change the excitability of MNs via GABA_A_ receptors involved in tonic inhibition.

### Functional implications: An orchestrated system mediated by glutamatergic and GABAergic synaptic inputs drives firing properties in oculomotor nucleus motoneurons

**Figure 8:**
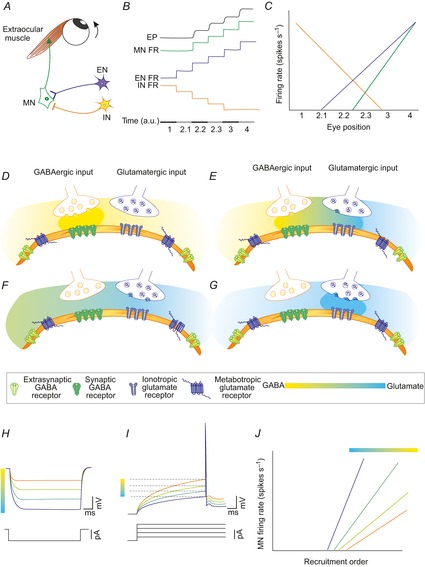
An orchestrated system mediated by glutamate- and GABA-synaptic inputs drives firing properties in ocular MNs *A*, showing EN and IN that provide glutamatergic and GABAergic inputs, respectively, to the MN. The firing frequency of the MN elicits the pattern of contraction of the extraocular muscle. *B*, FRs of IN and EN leading to discharge of the MN and altered EP. The bar corresponds to periods of time (in arbitrary units, a.u.) in which: (1) EN does not discharge, whereas IN is firing maximally; (2.1, 2.2, 2.3) EN and IN increase and decrease their FRs, respectively, in successive steps in the MN on-direction; (3,4) EN increases its FR in successive steps while IN does not fire. *C*, IN, EN and MN FRs during eye fixations represented in (*B*). *D–G*, release of GABA and glutamate from IN and EN, respectively. Receptors are in the MN surface membrane. These drawings correspond to the conditions indicated in scenarios 1–4 of (*B*) in this figure. The gradient from yellow to blue represents the levels of the two neurotransmitters in the synaptic cleft and non-synaptic space. *H*, reduction in the concentration of extracellular GABA leads to a decrease in the tonic inhibition and an increase in input resistance. *I*, increase in glutamate concentration leads to a reduction of the recruitment threshold current by reducing the action potential voltage threshold. *J*, concurrent effects on recruitment threshold and gain of a reduction in the GABA_A_-mediated tonic current and activation of metabotropic glutamate receptors. EN, excitatory neurons; EP, eye position; FR, firing rate; IN, inhibitory neurons; MNs, motoneurons.

A current hypothesis suggests that premotor synaptic drive to ocular MNs determines the positive covariation between recruitment threshold and eye position sensitivity in alert preparation (see Introduction). Premotor sources for the horizontal and vertical eye position signals originate in different regions of the brainstem, although they may be tightly coupled (Scudder *et al*. 2002). The abducens nucleus neurons receive inhibitory signals from the contralateral nucleus prepositus hypoglossi and the ipsilateral medial vestibular nucleus. These inhibitory position signals are balanced by excitatory input from the ipsilateral nucleus prepositus hypoglossi and contralateral medial vestibular nucleus (Scudder and Fuchs, 1992). The oculomotor and trochlear MNs receive input from the interstitial nucleus of Cajal that provide signals for vertical and torsional gaze-holding (Fukushima *et al*. 1992). The interstitial nucleus of Cajal contains inhibitory (Horn *et al*. 2003) and presumably excitatory (Ahlfeld *et al*. 2011) premotor neurons projecting to the MNs of vertical eye muscles. A vertical eye position signal is also delivered to MNs via neurons in the vestibular nuclei. Neurons lying in the ipsilateral superior vestibular nucleus provide the inhibitory signals, while neurons in the contralateral medial vestibular nucleus provide excitatory inputs (McCrea *et al*. 1987; Fukushima & Kaneko, 1995; Highstein & Holstein, 2006). Inhibitory inputs to oculomotor and trochlear MNs are mediated by GABA and excitatory inputs are mediated by glutamate (De la Cruz *et al*. 1992; Spencer & Baker, 1992; Wentzel *et al*. 1996; Nguyen & Spencer, 1999; Horn *et al*. 2003; Ying *et al*. 2008; Ahlfeld *et al*. 2011; Che Ngwa *et al*. 2014). Abducens neurons use glutamate as an excitatory transmitter (Nguyen & Spencer, 1999), while synaptic inhibition is mediated by GABA and glycine (Spencer *et al*. 1989; Lahjouji *et al*. 1996; Lorenzo *et al*. 2007). Premotor excitatory neurons increase their firing rate in the on-direction of MN activity, while premotor inhibitory afferents do the opposite (Fig.8*A*–*C*; Scudder *et al*. 2002; Sparks, 2002). Hence, ocular MN is driven in what has been termed a push–pull fashion by the two kinds of drive (Baker *et al*. 1981). These neurotransmitters act transiently during neuronal transmission and in a more persistent form to activate metabotropic glutamate receptors (Torres-Torrelo *et al*. 2012) and GABA_A_ receptor-mediated tonic current. We propose that the MN firing rate is essentially driven by transient neurotransmission. Metabotropic glutamate receptor and GABA_A_ receptor-mediated tonic current could act as systems of modulation refinement of the motor output of ocular MNs.

How do metabotropic glutamate receptors and GABA_A_ receptors mediate tonic current modulated MN activity? We propose that both receptor types work in an orchestrated manner. Thus, a rise in the extracellular concentration of glutamate would be accompanied by a proportional reduction of GABA levels (Fig.8*D*–*G*). In accordance with the effects of GABA (present work) and glutamate (Torres-Torrelo *et al*. 2012) in isolation, recruitment threshold of the MNs would decrease because of an increase in input resistance when ambient GABA concentration decreases (Fig.8*H*) and by a decrease in voltage threshold mediated by a gradual activation of metabotropic glutamate receptors (Torres-Torrelo *et al*. 2012; Fig.8*I*). These latter receptors also increase the firing rate gain (Young *et al*. 2004; Torres-Torrelo *et al*. 2012). The effects mediated by metabotropic glutamate receptors and GABA_A_ receptor-mediated tonic current would allow MNs to be recruited and for there to be an increased firing rate gain with reduced excitatory inputs (Fig.8*J*).

As has been reported, the negative covariation between threshold and *f–I* gain found in brain slices under control conditions disappears with glutamate treatment (Torres-Torrelo *et al*. 2012). As GABA did not affect gain, the source of positive covariation found in alert preparation remains uncertain. A possibility is that the concurrent effects of GABA and glutamate were more than those found separately. Hazel *et al*. (2002) in a neuronal simulation model for control of eye position that took the threshold and eye position sensitivity values from real recorded neurons within different premotor sources. Inhibitory input varied markedly with MN threshold, whereas excitatory inputs did not. Inhibitory synaptic drive to MNs was weak with the low-threshold eye position and strong with the high-threshold eye position. Under these conditions, the increase in inhibitory input led to an increase in threshold and eye position sensitivity and reproduces qualitatively the relationship between firing rate threshold and position sensitivity found experimentally in alert preparation. In agreement with this model, data reported here have shown an increase in tonic inhibition with the MN threshold, but the positive covariation between threshold and gain was not found. The input–output function is different when a single source is varied than when more than one synaptic input is concurrently active (Binder *et al*. 1993). Hence, further experimentation would be required to determine if the concurrent effects of GABA_A_ receptor-mediated tonic inhibition and excitatory metabotropic glutamate receptor activation would reproduce the *in vivo* result.

## Key points

- This study deals with synaptic mechanisms involved in the modulation of recruitment threshold and firing rate in ocular motoneurons. In particular, how high and low ambient GABA concentrations modulate these parameters.
- In a low ambient GABA concentration, ocular motoneurons exhibit a tonic GABA_A_ receptor-mediated current that modulates input resistance and recruitment threshold. The modulation effect increased with motoneuron size. Tonic inhibition shifts the firing frequency–current relationship without change in gain.
- Independent of cell size, a high ambient GABA concentration reduced input resistance and increased motoneuron recruitment threshold without a change in firing frequency gain.
- Our results demonstrate that GABA_A_ mediated tonic inhibition and glutamate mediated current modulate recruitment threshold and firing behaviour of ocular motoneurons depending on motoneuron size. These synaptic mechanisms, dependent on cell size, could be underlying the positive relationship between threshold and eye position sensitivity reported in ocular motoneurons recorded in alert preparation.

## References

[b1] Ahlfeld J, Mustari M, Horn AK (2011). Sources of calretinin inputs to motoneurons of extraocular muscles involved in upgaze. Ann N Y Acad Sci.

[b2] Baker R, Evinger C, McCrea RA (1981). Some thoughts about the three neurons in the vestibular ocular reflex. Ann N Y Acad Sci.

[b3] Bianchi L, Ballini C, Colivicchi MA, Della Corte L, Giovannini MG, Pepeu G (2003). Investigation on acetylcholine, aspartate, glutamate and GABA extracellular levels from ventral hippocampus during repeated exploratory activity in the rat. Neurochem Res.

[b86] Binder MD, Heckman CJ, Powers RK (1993). How different afferent inputs control motoneuron discharge and the output of the motoneuron pool. Curr Opin Neurobiol.

[b4] Binder MD, Robinson FR, Powers RK (1998). Distribution of effective synaptic currents in cat triceps surae motoneurons. VI. Contralateral pyramidal tract. J Neurophysiol.

[b5] Brickley SG, Cull-Candy SG, Farrant M (1996). Development of a tonic form of synaptic inhibition in rat cerebellar granule cells resulting from persistent activation of GABA_A_ receptors. J Physiol.

[b6] Brizzi L, Meunier C, Zytnicki D, Donnet M, Hansel D, Lamotte D'Incamps B, Van Vreeswijk C (2004). How shunting inhibition affects the discharge of lumbar motoneurones: a dynamic clamp study in anaesthetized cats. J Physiol.

[b7] Broussard DM, DeCharms RC, Lisberger SG (1995). Inputs from the ipsilateral and contralateral vestibular apparatus to behaviorally characterized abducens neurons in rhesus monkeys. J Neurophysiol.

[b8] Carrascal L, Nieto-Gonzalez JL, Núñez-Abades P, Torres B (2006). Temporal sequence of changes in electrophysiological properties of oculomotor motoneurons during postnatal development. Neuroscience.

[b9] Carvalho TP, Buonomano DV (2009). Differential effects of excitatory and inhibitory plasticity on synaptically driven neuronal input-output functions. Neuron.

[b10] Castro A, Aguilar J, Andrés C, Felix R, Delgado-Lezama R (2011). GABA_A_ receptors mediate motoneuron tonic inhibition in the turtle spinal cord. Neuroscience.

[b11] Chadderton P, Margrie TW, Häusser M (2004). Integration of quanta in cerebellar granule cells during sensory processing. Nature.

[b12] Chance FS, Abbott LF, Reyes AD (2002). Gain modulation from background synaptic input. Neuron.

[b13] Che Ngwa E, Zeeh C, Messoudi A, Büttner-Ennever JA, Horn AK (2014). Delineation of motoneuron subgroups supplying individual eye muscles in the human oculomotor nucleus. Front Neuroanat.

[b14] Chesnoy-Marchais D (2013). Bicuculline- and neurosteroid-sensitive tonic chloride current in rat hypoglossal motoneurons and atypical dual effect of SR95531. Eur J Neurosci.

[b15] Cope TC, Sokoloff AJ (1999). Orderly recruitment among motoneurons supplying different muscles. J Physiol Paris.

[b16] Davis-López de Carrizosa MA, Morado-Díaz CJ, Miller JM, de la Cruz RR, Pastor AM (2011). Dual encoding of muscle tension and eye position by abducens motoneurons. J Neurosci.

[b17] de la Cruz RR, Pastor AM, Martínez-Guijarro FJ, López-García C, Delgado-García JM (1992). Role of GABA in the extraocular motor nuclei of the cat: a postembedding immunocytochemical study. Neuroscience.

[b18] Dean P (1997). Simulated recruitment of medial rectus motoneurons by abducens internuclear neurons: synaptic specificity *vs*. intrinsic motoneuron properties. J Neurophysiol.

[b19] Delgado-Garcia JM, del Pozo F, Baker R (1986). Behavior of neurons in the abducens nucleus of the alert cat–I. Motoneurons. Neuroscience.

[b20] Duguid I, Branco T, London M, Chadderton P, Häusser M (2012). Tonic inhibition enhances fidelity of sensory information transmission in the cerebellar cortex. J Neurosci.

[b21] Duque D, Malmierca MS, Caspary DM (2014). Modulation of stimulus-specific adaptation by GABA_A_ receptor activation or blockade in the medial geniculate body of the anesthetized rat. J Physiol.

[b22] Farrant M, Nusser Z (2005). Variations on an inhibitory theme: phasic and tonic activation of GABA_A_ receptors. Nat Rev Neurosci.

[b23] Feldblum S, Erlander MG, Tobin AJ (1993). Different distributions of GAD65 and GAD67 mRNAs suggest that the two glutamate decarboxylases play distinctive functional roles. J Neurosci Res.

[b24] Fuchs AF, Luschei ES (1970). Firing patterns of abducens neurons of alert monkeys in relationship to horizontal eye movement. Neurophysiol.

[b25] Fuchs AF, Scudder CA, Kaneko CR (1988). Discharge patterns and recruitment order of identified motoneurons and internuclear neurons in the monkey abducens nucleus. J Neurophysiol.

[b26] Fukushima K, Kaneko CR (1995). Vestibular integrators in the oculomotor system. Neurosci Res.

[b87] Fukushima K, Kaneko CR, Fuchs AF (1992). The neuronal substrate of integration in the oculomotor system. Prog Neurobiol.

[b27] Gao H, Smith BN (2010). Tonic GABA_A_ receptor-mediated inhibition in the rat dorsal motor nucleus of the vagus. J Neurophysiol.

[b28] Glykys J, Mody I (2007). Activation of GABA_A_ receptors: views from outside the synaptic cleft. Neuron.

[b29] Grantyn R, Grantyn A (1978). Morphological and electrophysiological properties of cat abducens motoneurons. Exp Brain Res.

[b30] Hamann M, Rossi DJ, Attwell D (2002). Tonic and spillover inhibition of granule cells control information flow through cerebellar cortex. Neuron.

[b31] Hazel TR, Sklavos SG, Dean P (2002). Estimation of premotor synaptic drives to simulated abducens motoneurons for control of eye position. Exp Brain Res.

[b32] Heckman CJ, Binder MD (1988). Analysis of effective synaptic currents generated by homonymous Ia afferent fibers in motoneurons of the cat. J Neurophysiol.

[b33] Heckman CJ, Binder MD (1991). Analysis of Ia-inhibitory synaptic input to cat spinal motoneurons evoked by vibration of antagonist muscles. J Neurophysiol.

[b34] Heckman CJ, Binder MD (1993). Computer simulations of motoneuron firing rate modulation. J Neurophysiol.

[b35] Henneman E, Somjen G, Carpenter DO (1965). Functional significance of cell size in spinal motoneurons. J Neurophysiol.

[b36] Highstein SM (1973). Synaptic linkage in the vestibulo-ocular and cerebello-vestibular pathways to the VIth nucleus in the rabbit. Exp Brain Res.

[b37] Highstein SM, Holstein GR (2006). The anatomy of the vestibular nuclei. Prog Brain Res.

[b38] Horn AK, Helmchen C, Wahle P (2003). GABAergic neurons in the rostral mesencephalon of the macaque monkey that control vertical eye movements. Ann N Y Acad Sci.

[b39] Ingham NJ, McAlpine D (2005). GABAergic inhibition controls neural gain in inferior colliculus neurons sensitive to interaural time differences. J Neurosci.

[b40] Ito M, Highstein SM, Tsuchiya T (1970). The postsynaptic inhibition of rabbit oculomotor neurones by secondary vestibular impulses and its blockage by picrotoxin. Brain Res.

[b41] Kersanté F, Rowley SC, Pavlov I, Gutièrrez-Mecinas M, Semyanov A, Reul JM, Walker MC, Linthorst AC (2013). A functional role for both g-aminobutyric acid (GABA) transporter-1 and GABA transporter-3 in the modulation of extracellular GABA and GABAergic tonic conductances in the rat hippocampus. J Physiol.

[b42] Kullmann DM, Ruiz A, Rusakov DM, Scott R, Semyanov A, Walker MC (2005). Presynaptic, extrasynaptic and axonal GABA_A_ receptors in the CNS: where and why?. Prog Biophys Mol Biol.

[b43] Lahjouji F, Barbe A, Chazal G, Bras H (1996). Evidence for colocalization of GABA and glycine in afferents to retrogradely labelled rat abducens motoneurones. Neurosci Lett.

[b44] Lorenzo LE, Russier M, Barbe A, Fritschy JM, Bras H (2007). Differential organization of g-aminobutyric acid type A and glycine receptors in the somatic and dendritic compartments of rat abducens motoneurons. J Comp Neurol.

[b45] Mann EO, Paulsen O (2007). Role of GABAergic inhibition in hippocampal network oscillations. Trends Neurosci.

[b46] Margeta-Mitrovic M, Mitrovic I, Riley RC, Jan LY, Basbaum AI (1999). Immunohistochemical localization of GABA_B_ receptors in the rat central nervous system. J Comp Neurol.

[b47] McCrea RA, Strassman A, Highstein SM (1987). Anatomical and physiological characteristics of vestibular neurons mediating the vertical vestibulo-ocular reflexes of the squirrel monkey. J Comp Neurol.

[b48] Mendell LM (2005). The size principle: a rule describing the recruitment of motoneurons. J Neurophysiol.

[b49] Mitchell SJ, Silver RA (2003). Shunting inhibition modulates neuronal gain during synaptic excitation. Neuron.

[b50] Mortensen M, Kristiansen U, Ebert B, Frølund B, Krogsgaard-Larsen P, Smart TG (2004). Activation of single heteromeric GABA_A_ receptor ion channels by full and partial agonists. J Physiol.

[b51] Nguyen LT, Spencer RF (1999). Abducens internuclear and ascending tract of Deiters inputs to medial rectus motoneurons in the cat oculomotor nucleus: neurotransmitters. J Comp Neurol.

[b52] Nieto-Gonzalez JL, Carrascal L, Nunez-Abades P, Torres B (2007). Phasic and tonic firing properties in rat oculomotor nucleus motoneurons, studied in vitro. Eur J Neurosci.

[b53] Numata JM, van Brederode JF, Berger AJ (2012). Lack of an endogenous GABA_A_ receptor-mediated tonic current in hypoglossal motoneurons. J Physiol.

[b54] Nusser Z, Mody I (2002). Selective modulation of tonic and phasic inhibitions in dentate gyrus granule cells. J Neurophysiol.

[b55] Nusser Z, Roberts JD, Baude A, Richards JG, Somogyi P (1995). Relative densities of synaptic and extrasynaptic GABA_A_ receptors on cerebellar granule cells as determined by a quantitative immunogold method. J Neurosci.

[b56] Ortinski PI, Turner JR, Barberis A, Motamedi G, Yasuda RP, Wolfe BB, Kellar KJ, Vicini S (2006). Deletion of the GABA_A_ receptor a1 subunit increases tonic GABA_A_ receptor current: a role for GABA uptake transporters. J Neurosci.

[b57] Park JB, Skalska S, Stern JE (2006). Characterization of a novel tonic g-aminobutyric acid_A_ receptor-mediated inhibition in magnocellular neurosecretory neurons and its modulation by glia. Endocrinology.

[b58] Pastor AM, Gonzalez-Forero D (2003). Recruitment order of cat abducens motoneurons and internuclear neurons. J Neurophysiol.

[b59] Pavlov I, Savtchenko LP, Kullmann DM, Semyanov A, Walker MC (2009). Outwardly rectifying tonically active GABA_A_ receptors in pyramidal cells modulate neuronal offset, not gain. J Neurosci.

[b60] Petrini EM, Marchionni I, Zacchi P, Sieghart W, Cherubini E (2004). Clustering of extrasynaptic GABA_A_ receptors modulates tonic inhibition in cultured hippocampal neurons. J Biol Chem.

[b61] Pouille F, Scanziani M (2001). Enforcement of temporal fidelity in pyramidal cells by somatic feed-forward inhibition. Science.

[b62] Powers RK, Robinson FR, Konodi MA, Binder MD (1993). Distribution of rubrospinal synaptic input to cat triceps surae motoneurons. J Neurophysiol.

[b63] Precht W, Baker R, Okada Y (1973). Evidence for GABA as the synaptic transmitter of the inhibitory vestibulo-ocular pathway. Exp Brain Res.

[b64] Richerson GB, Wu Y (2003). Dynamic equilibrium of neurotransmitter transporters: not just for reuptake anymore. J Neurophysiol.

[b65] Rothman JS, Cathala L, Steuber V, Silver RA (2009). Synaptic depression enables neuronal gain control. Nature.

[b66] Ruiz A, Fabian-Fine R, Scott R, Walker MC, Rusakov DA, Kullmann DM (2003). GABA_A_ receptors at hippocampal mossy fibers. Neuron.

[b67] Russier M, Kopysova IL, Ankri N, Ferrand N, Debanne D (2002). GABA and glycine co-release optimizes functional inhibition in rat brainstem motoneurons *in vitro*. J Physiol.

[b68] Scimemi A, Semyanov A, Sperk G, Kullmann DM, Walker MC (2005). Multiple and plastic receptors mediate tonic GABA_A_ receptor currents in the hippocampus. J Neurosci.

[b69] Scudder CA, Fuchs AF (1992). Physiological and behavioral identification of vestibular nucleus neurons mediating the horizontal vestibuloocular reflex in trained rhesus monkeys. J Neurophysiol.

[b70] Scudder CA, Kaneko CS, Fuchs AF (2002). The brainstem burst generator for saccadic eye movements: a modern synthesis. Exp Brain Res.

[b71] Sebe JY, van Brederode JF, Berger AJ (2006). Inhibitory synaptic transmission governs inspiratory motoneuron synchronization. J Neurophysiol.

[b72] Semyanov A, Walker MC, Kullmann DM (2003). GABA uptake regulates cortical excitability via cell type-specific tonic inhibition. Nat Neurosci.

[b73] Semyanov A, Walker MC, Kullmann DM, Silver RA (2004). Tonically active GABA_A_ receptors: modulating gain and maintaining the tone. Trends Neurosci.

[b74] Soghomonian JJ, Pinard R, Lanoir J (1989). GABA innervation in adult rat oculomotor nucleus: a radioautographic and immunocytochemical study. J Neurocytol.

[b75] Sparks DL (2002). The brainstem control of saccadic eye movements. Nat Rev Neurosci.

[b76] Spencer RF, Baker R (1992). GABA and glycine as inhibitory neurotransmitters in the vestibuloocular reflex. Ann N Y Acad Sci.

[b77] Spencer RF, Wang SF (1996). Immunohistochemical localization of neurotransmitters utilized by neurons in the rostral interstitial nucleus of the medial longitudinal fasciculus (riMLF) that project to the oculomotor and trochlear nuclei in the cat. J Comp Neurol.

[b88] Spencer RF, Wang SF, Baker R (1992). The pathways and functions of GABA in the oculomotor system. Prog Brain Res.

[b78] Spencer RF, Wenthold RJ, Baker R (1989). Evidence for glycine as an inhibitory neurotransmitter of vestibular, reticular, and prepositus hypoglossi neurons that project to the cat abducens nucleus. J Neurosci.

[b79] Stórustovu S, Ebert B (2006). Pharmacological characterization of agonists at d-containing GABA_A_ receptors: Functional selectivity for extrasynaptic receptors is dependent on the absence of g_2_. J Pharmacol Exp Ther.

[b80] Torres-Torrelo J, Rodríguez-Rosell D, Nunez-Abades P, Carrascal L, Torres B (2012). Glutamate modulates the firing rate in oculomotor nucleus motoneurons as a function of the recruitment threshold current. J Physiol.

[b81] Walker MC, Semyanov A (2008). Regulation of excitability by extrasynaptic GABA_A_ receptors. Results Probl Cell Differ.

[b82] Wentzel PR, Gerrits NM, de Zeeuw CI (1996). GABAergic and glycinergic inputs to the rabbit oculomotor nucleus with special emphasis on the medial rectus subdivision. Brain Res.

[b83] Westcott SL, Powers RK, Robinson FR, Binder MD (1995). Distribution of vestibulospinal synaptic input to cat triceps surae motoneurons. Exp Brain Res.

[b84] Ying HS, Fackelmann K, Messoudi A, Tang XF, Büttner-Ennever JA, Horn AK (2008). Neuronal signalling expression profiles of motoneurons supplying multiply or singly innervated extraocular muscle fibres in monkey. Prog Brain Res.

[b85] Young SR, Chuang SC, Wong RK (2004). Modulation of afterpotentials and firing pattern in guinea pig CA3 neurones by group I metabotropic glutamate receptors. J Physiol.

